# Alterations in Glutathione Redox Metabolism, Oxidative Stress, and Mitochondrial Function in the Left Ventricle of Elderly Zucker Diabetic Fatty Rat Heart

**DOI:** 10.3390/ijms131216241

**Published:** 2012-11-30

**Authors:** Haider Raza, Annie John, Frank C. Howarth

**Affiliations:** 1 Department of Biochemistry, College of Medicine and Health Sciences, United Arab Emirates University, Al Ain, P.O. Box 17666, UAE; E-Mail: anniej@uaeu.ac.ae; 2 Department of Physiology, College of Medicine and Health Sciences, United Arab Emirates University, Al Ain, P.O. Box 17666, UAE; E-Mail: chris.howarth@uaeu.ac.ae

**Keywords:** Zucker rats, diabetes, obesity, oxidative stress, cardiomyocytes, mitochondria

## Abstract

The Zucker diabetic fatty (ZDF) rat is a genetic model in which the homozygous (FA/FA) male animals develop obesity and type 2 diabetes. Morbidity and mortality from cardiovascular complications, due to increased oxidative stress and inflammatory signals, are the hallmarks of type 2 diabetes. The precise molecular mechanism of contractile dysfunction and disease progression remains to be clarified. Therefore, we have investigated molecular and metabolic targets in male ZDF (30–34 weeks old) rat heart compared to age matched Zucker lean (ZL) controls. Hyperglycemia was confirmed by a 4-fold elevation in non-fasting blood glucose (478.43 ± 29.22 mg/dL in ZDF *vs.* 108.22 ± 2.52 mg/dL in ZL rats). An increase in reactive oxygen species production, lipid peroxidation and oxidative protein carbonylation was observed in ZDF rats. A significant increase in CYP4502E1 activity accompanied by increased protein expression was also observed in diabetic rat heart. Increased expression of other oxidative stress marker proteins, HO-1 and iNOS was also observed. GSH concentration and activities of GSH-dependent enzymes, glutathione *S*-transferase and GSH reductase, were, however, significantly increased in ZDF heart tissue suggesting a compensatory defense mechanism. The activities of mitochondrial respiratory enzymes, Complex I and Complex IV were significantly reduced in the heart ventricle of ZDF rats in comparison to ZL rats. Western blot analysis has also suggested a decreased expression of IκB-α and phosphorylated-JNK in diabetic heart tissue. Our results have suggested that mitochondrial dysfunction and increased oxidative stress in ZDF rats might be associated, at least in part, with altered NF-κB/JNK dependent redox cell signaling. These results might have implications in the elucidation of the mechanism of disease progression and designing strategies for diabetes prevention.

## 1. Introduction

Cardiomyopathy and other cardiovascular complications associated with increased inflammatory and oxidative stress responses are the major causes of accelerated atherosclerosis, obesity and diabetes [1–3]. Persistent hyperglycemia and hyperlipidemia are believed to be the main causes of increased oxidative stress, mitochondrial dysfunctions, fibrosis and apoptosis of cardiomyocytes in diabesity and associated complications [4–6].

The male Zucker diabetic fatty (ZDF) rat is a genetic model in which homozygous (FA/FA) animals spontaneously develop type 2 diabetes and obesity, whereas female rats become diabetic only after feeding high fat diet [7]. The main reason for this resistance in female Zucker rats appears to be the presence of high concentration of antioxidant glutathione (GSH) and low oxidative stress [8]. ZDF rats also exhibit increased insulin resistance, oxidative stress, hyperlipidemia, increased inflammatory responses and abnormal energy metabolism which are the key features of diabesity. There are studies which suggest that the attenuation of oxidative stress by treatment with antioxidant therapeutic drugs or dietary products have normalized the glycemic index and metabolic complications in diabesity [9,10]. Our previous studies, using both type 1 and type 2 diabetic models, have also suggested that myocardial Ca^2+^ signaling genes and proteins have also been drastically affected in diabetes and obesity related complications [11–14]. However, in these studies, it is not clear if these changes are in response to increased oxidative stress due to increased reactive oxygen species (ROS) and/or decreased GSH antioxidant metabolism. Our previous studies, using *in vivo* type 1 diabetes models have strongly suggested increased oxidative stress, mitochondrial dysfunction and compromised energy and GSH metabolism in chronic diabetic complications [15–18]. Our present aim is, therefore, to investigate the role of oxidative stress, GSH-dependent antioxidant metabolism and mitochondrial functions in isolated cardiac myocytes from left ventricle of ZDF rats and to compare them with age matched Zucker lean (ZL) rats. Our results have provided a better insight of the etiology and pathology of diabetes and obesity associated complications and have implications in designing therapeutic approaches.

## 2. Results and Discussion

### 2.1. Alterations in Oxidative Stress in Zucker Diabetic Rat Hearts

**Figure 1:**
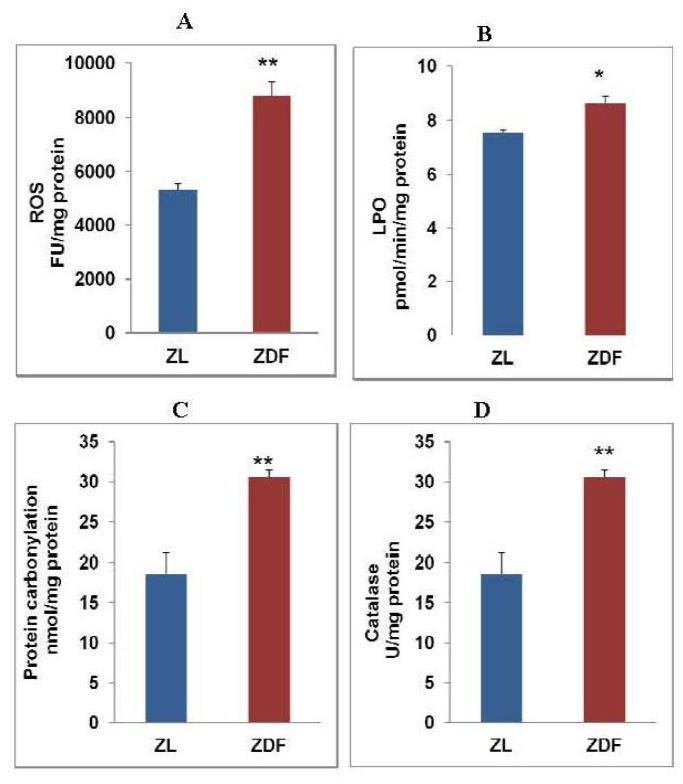
Reactive oxygen species (ROS), lipid peroxidation (LPO), protein carbonylation and catalase activity in Zucker diabetic fatty (ZDF) rat heart. ROS (**A**) was measured fluorimetrically as described in Experimental section. NADPH-dependent total LPO (**B**), DNPH-coupled protein peroxidation (**C**) and catalase (**D**) were measured as described [15–19]. Results are expressed as mean ± S.E.M. from three independent experiments and asterisks indicate significant difference (^*^p < 0.05 and ^**^p < 0.01).

An increase in oxidative stress in diabetic heart myocytes was observed as shown in Figure 1A–D. An increase in ROS production (Figure 1A) was accompanied by a moderate but significant increase in LPO (Figure 1B) and an increase in oxidative protein carbonylation as observed by DNPH coupling of oxidized proteins (Figure 1C). Catalase activity was found to be significantly activated (Figure 1D). These results suggest that catalase appears to be the main enzyme involved in H_2_O_2_ clearance as we observed no marked alteration in GSH-Px activity in ZDF rats.

### 2.2. Alterations in GSH Metabolism in Zucker Diabetic Rat Hearts

**Figure 2:**
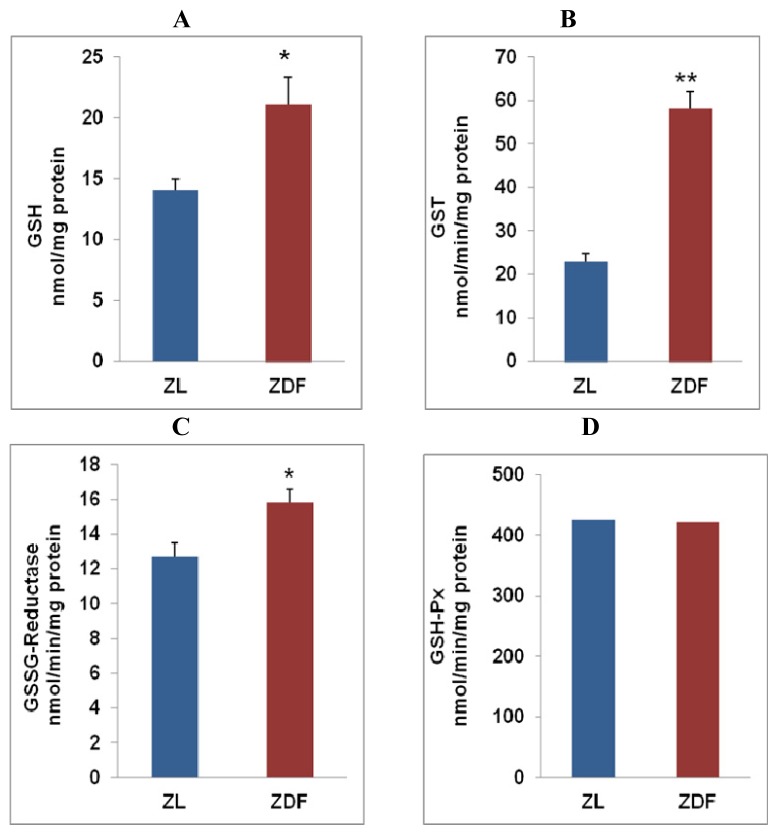
Glutathione (GSH) concentration and GSH metabolism in ZDF rat heart: Total free GSH (**A**) and GSH metabolizing enzymes; glutathione *S*-transferase (GST) (**B**), glutathione-reductase (GSSG-reductase) (**C**) and glutathione peroxidase (GSH-Px) (**D**) were measured in total cardiac myocyte homogenate from left ventricles as described [15,16]. Results are expressed as mean ± S.E.M. from three independent experiments and asterisks indicate significant difference (^*^p < 0.05 and ^**^p < 0.01).

As shown in Figure 2, GSH concentration was markedly (~2-fold) increased (Figure 2A) in ZDF rat myocytes compared to ZL rat heart. Similarly, a profound increase in GSH-CDNB conjugating activity by GST enzymes was also observed (Figure 2B). On the other hand, there was no significant increase in GSH-Px activity (Figure 2D) while GSSG-reductase activity was found to be significantly increased (Figure 2C). These results suggest that GSH-dependent antioxidant defense mechanisms have been activated in ZDF rat hearts. An increase in GSH concentration and GSH-dependent conjugation of ROS in ZDF rat heart and increased regeneration of reduced GSH by GSSG-reductase might be involved in protecting the cardiac myocytes from oxidative damage.

### 2.3. Induction of CYP450 Activity in Zucker Diabetic Rat Hearts

**Figure 3:**
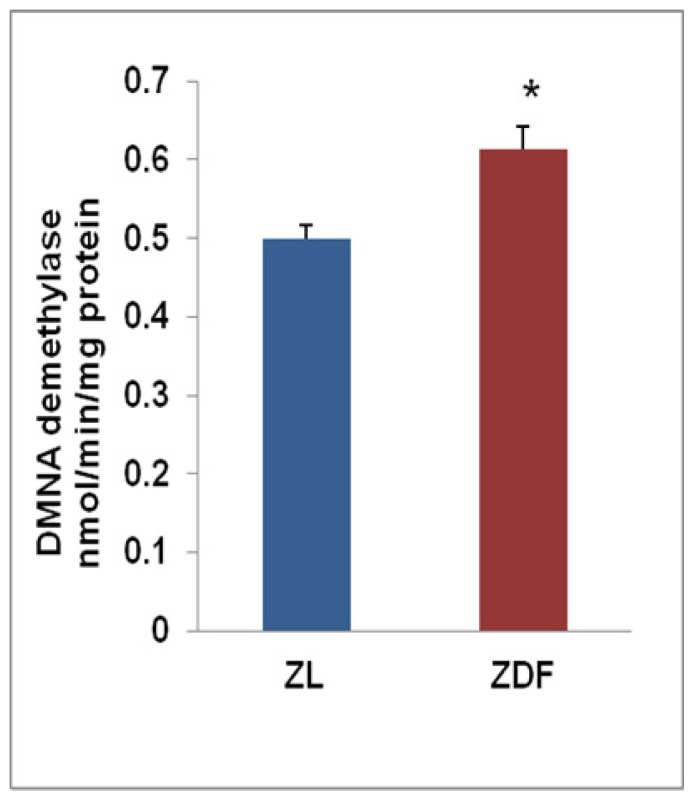
Cytochrome P450 2E1 (CYP 2E1) activity in ZDF rat heart: CYP 2E1 activity in ZDF and Zucker lean (ZL) rat heart left ventricle fraction was measured using dimethylnitrosamine (DMNA) as a substrate as described before [16]. Results are expressed as mean ± S.E.M. from three independent experiments and asterisk (^*^) indicates significant difference (*p* < 0.05).

Cytochrome P450 2E1 (CYP 2E1) enzyme activity was also increased significantly in ZDF rat heart when compared to ZL rat heart myocytes (Figure 3). CYP 2E1 isoenzyme has been reported to be involved in oxidative stress and an increase in enzyme activity has also been reported in diabetes and obesity [16,20,21].

### 2.4. Alterations in Mitochondrial Respiratory Functions in ZDF Rat Hearts

**Figure 4:**
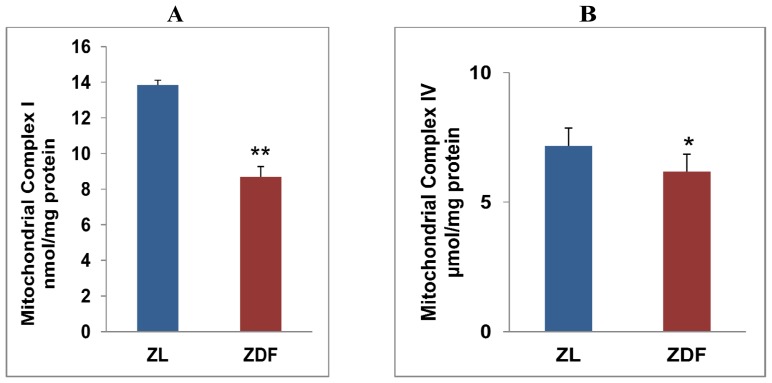
Mitochondrial respiratory Complex I and IV activities in ZDF rat heart: Mitochondrial respiratory enzymes complexes, Complex I (**A**) and Complex IV (**B**) were measured in freshly prepared ventricular fractions using appropriate substrates as described in the Experimental section. Results are expressed as mean ± S.E.M. from three independent experiments and asterisks indicate significant difference (^*^p < 0.05 and ^**^p < 0.01).

A significant decrease in the activities of mitochondrial inner membrane respiratory complexes has been observed in ZDF rat hearts (Figure 4). NADH-dependent ubiquinone oxidoreductase (Complex I) activity was markedly reduced (42%) in ZDF rat heart myocytes when compared to ZL rat hearts (Figure 4A). Similarly, a significant decrease in activity of the terminal respiratory enzyme, cytochrome c oxidase (Complex IV), was also observed in ZDF rat hearts (Figure 4B). These results have clearly suggested that mitochondrial bioenergetics (ATP production) is affected in the cardiac myocytes of ZDF rats.

### 2.5. Alteration in the Expression of Oxidative Stress Marker and Transcription Regulatory Proteins in ZDF Rat Hearts

**Figure 5:**
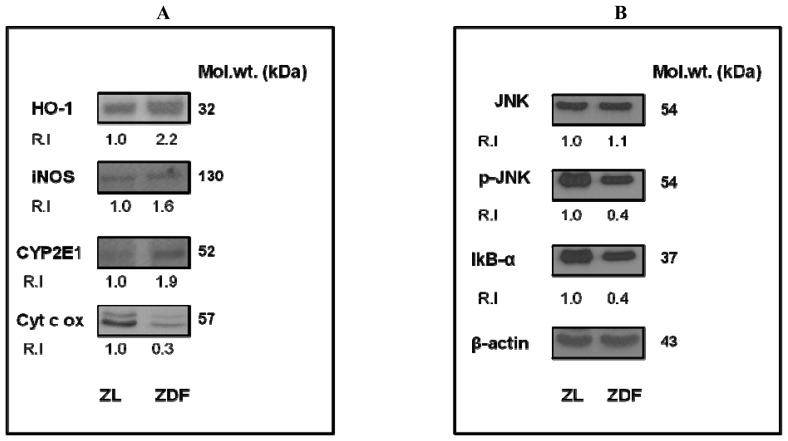
Expression of oxidative stress and cell signaling marker enzymes/proteins in ZDF rat heart. Protein (50 μg) from ZDF and ZL rat hearts were subjected to 12% sodium dodecylsulphate polyacrylamide gel electrophoresis (SDS-PAGE) [22] and Western blot analyses [23] to visualize immunoreactivity of specific marker proteins for oxidative stress (**A**: HO-1, iNOS, CYP2E1 and Cyt c ox) and cell signaling (**B**: JNK, p-JNK and IκB-α). β-actin was used as a loading control. R.I values indicate relative intensity (of the protein band) using expression of the proteins in ZL as 1.0. The figures are representative of 2–3 experiments. Molecular weights shown are in kDa.

As shown in Figure 5, SDS-PAGE and Western blot analyses have demonstrated increased expression of oxidative stress marker proteins, HO-1, iNOS and CYP 2E1 (Figure 5A). On the other hand the expression of cytochrome c oxidase (Cyt c ox) enzyme subunit 1 was found to be markedly reduced suggesting inhibition of mitochondrial energy metabolism under increased oxidative stress conditions as seen in ZDF rats. We also observed a reduced expression of phosphorylated JNK (p-JNK) and IκB-α while no apparent change in the expression of JNK (non-phosphorylated) was noticed (Figure 5B). These results suggest alteration in cell signaling/transcription regulation in ZDF rat heart under oxidative stress conditions when compared to ZL rat hearts.

### 2.6. Discussion

Our previous studies in younger (9–13 weeks) animals, the ZDF rats weighed significantly more than controls [13]. In the present study, experiments were performed in male animals when they were 30–34 weeks of age. Bodyweight in the ZDF rats was not significantly different from controls. Diabetes mellitus was characterized by a 4-fold increase in blood glucose. Heart function is compromised in the ZDF rats. With age, the severity of diabetes and its complications worsens and ZDF rats are likely to become more reliant on the use of lipids and lipid reserves to meet metabolic requirements. This may partly account for reduced weight in aged ZDF rats. Recently, using the same cohort of elderly Zucker diabetic rats, it was demonstrated that ventricular myocyte function was well preserved in ZDF rat heart [14]. Although resting cell length was reduced, the amplitude and time course of myocyte shortening was not altered in ZDF rat compared to controls. Ventricular myocyte shortening was associated with altered Ca^++^ transport. However, there was no significant difference in the ratio of heart weight to body weight. A recent study in ZDF rats [24] suggests that diabetes per se is not a critical factor in the induction of clinically significant cardiac dysfunction and some other factors related to obesity might have greater impact on cardiac function. Previous studies in other experimental models of diabetes, for example, streptozotocin-induced diabetic rats, have demonstrated reduced weight gain which was variously associated with hyperglycemia, hypoinsulinemia, glycosuria, depletion of body fat and liver glycogen [25,26]. A previous study demonstrated a prolonged time course of myocyte shortening and relaxation of shortening in 9–13-week ZDF rats [13]. As in obese humans, ZDF rats exhibit early β-cell compensation (hyperplasia) of insulin resistance followed by decompensation (loss of cells) [27]. The early changes in β-cell responsiveness to glucose may contribute to the hyperinsulinemia and subsequent insulin resistance [28]. ZDF rats also exhibit reduced heart rate [29].

In type 2 diabetes and obesity metabolic disorders and inflammatory-linked pathologies, as seen in ZDF rats, an increased production of reactive oxygen species, oxidative stress, altered cell signaling and mitochondrial dysfunctions in heart and other tissues were observed [3,30–33]. We have also confirmed increased oxidative stress due to increased production of ROS, resulting in increased oxidative lipid and protein peroxidation in ZDF rat cardiac myocytes. Catalase appears to be the main enzyme involved in H_2_O_2_ clearance as we observed no marked alteration in GSH-Px activity while catalase activity was markedly increased in ZDF rats. Activation of catalase in ZDF rat heart might reduce the pool of H_2_O_2_*in vivo*, and hence disturb the balance of metabolism of this stable ROS which might have implications in insulin signaling. There are reports which suggest that over expression of antioxidant enzymes and altered H_2_O_2_ clearance in obesity may be responsible for the development of insulin resistance and interfere in insulin-dependent signaling [34]. Activation of GSH metabolism by GST enzyme and recycling of GSH by GSSG-reductase appears to be a defense response to prevent the overquenching of intracellular ROS required for insulin signaling. H_2_O_2_ has also been shown to modulate nitric oxide synthesis in cardiomyocytes [35]. Our study has clearly indicated that the regeneration of GSH from GSSG significantly increased in ZDF rat heart due to an increase in GSSG-reductase activity while no significant alteration in GSH-peroxidase (which utilizes GSSG) activity was observed. This was accompanied by an increased GSH pool in cardiac myocytes. Our results thus suggest an induction of GSH-dependent antioxidant adaptive response in cardiac myocytes in ZDF rat heart.

We observed an increased expression of CYP 2E1, iNOS, HO-1 accompanied by increased protein carbonylation in ZDF rat heart suggesting increased oxidative stress. Our previous studies on streptozotocin-induced chronic metabolic complications have also shown increased oxidative stress, mitochondrial dysfunction and altered expression of CYP 2E1 and oxidative stress marker proteins [16–18]. Increased expression of CYP 2E1 in oxidative stress conditions have also been shown to induce apoptosis in cardiomyocytes [36]. Recently, it has also been shown that the inhibition of protein carbonylation by antioxidants prevents the metabolic complications in ZDF rats [37]. Mitochondrial dysfunction, reduction in the activities of the respiratory complexes and reduced expression of cytochrome c oxidase was also observed in ZDF rat heart. Altered oxygen consumption in ZDF rat heart might be associated with compromised mitochondrial bioenergetics, which resulted in the reduced activities of the respiratory complexes. Altered mitochondrial function and increased oxidative stress in ZDF rats has consequences in modulating JNK and NF-κB dependent cell signaling. We observed decreased JNK phosphorylation and reduced expression of IκB-α in ZDF rat heart. This might suggest an increased inflammatory response in ventricular myocytes. A recent study has also shown a decreased expression of p-JNK in transgenic type 2 diabetic rats which is responsible for modulation of MAPK cascade as observed in diabetic cardiomyopathy [38]. JNK-dependent activation of NF-κB in cardiomyocytes induced by hyperglycemia, inflammation and oxidative stress has also been reported [39]. It has also been suggested that activation of JNK can be negatively regulated by NF-κB inhibition [40]. Altered JNK-dependent signaling pathway, due to increased oxidative stress, has also been reported in human islets and ZDF rats leading to the onset of mitochondrial dysfunction in the diabetic islets [41]. Increased NF-κB activity and decrease in the inhibitory IκB-α expression also suggest an increase in proinflammatory signaling in obesity [42]. Increased NF-κB activity as seen in obesity and other inflammatory conditions is also involved in uncoupling insulin resistance from lipid metabolism [43]. A study by Aragno *et al.*[44] has also shown reduced left ventricle myocardial contractility and increased cardiomyopathy, following the impairment of NF-κB signaling in ZDF rats. Our results may also suggest a crucial role of these signaling proteins in cardiomyocyte survival from oxidative stress related proinflammatory responses.

## 3. Experimental Section

### 3.1. Chemicals

Cytochrome c, reduced and oxidized glutathione (GSH), 5,5′-dithio-bis(2-nitrobenzoic acid), 1-chloro 2,4-dinitrobenzene (CDNB), cumene hydroperoxide, glutathione reductase, thiobarbituric acid, NADH, NADPH, coenzyme Q2, antimycin A, dodecyl maltoside, dimethylnitrosamine (DMNA), dimethylphenylhydrazine (DNPH) were purchased from Sigma-Aldrich Fine Chemicals (St. Louis, MO, USA). 2′,7′-Dichlorofluorescein diacetate (DCFDA) was procured from Molecular Probes (Eugene, OR, USA). Polyclonal antibodies against iNOS, CYP2E1, cytochrome c oxidase subunit 1, JNK, IκB-α and β-actin were purchased from Santa Cruz Biotechnology Inc. (Santa Cruz, CA, USA), HO-1 from Abcam (Cambridge, MA, USA) and p-JNK from Cell Signaling Technology, Inc. (Danvers, MA, USA). Reagents for SDS-PAGE and Western blot analyses were purchased from Gibco BRL (Grand Island, NY, USA) and Bio Rad Laboratories (Richmond, CA, USA).

### 3.2. Animal and Tissue Preparation

Experiments were performed in elderly (aged 30–34 weeks) Zucker diabetic fatty (ZDF; FA/FA) rats (*n* = 4, average body *wt* = 485 g; average blood glucose = 478 mg/dL) and age-matched Zucker lean (ZL; +/FA) controls (*n* = 5, average body *wt* = 400 g; average blood glucose = 108 mg/dL) (Charles River Laboratories, UK). Approval for this project was obtained from the Animal Ethics Research Committee, College of Medicine & Health Sciences, United Arab Emirates University and all the animals were used according to the safe practice for animals in research guidelines as stipulated by NIH, USA.

Left ventricle heart muscles were dissected from male ZDF and from age-matched ZL control rats and rinsed with ice-cold saline. Isolated tissues were homogenized (10% *w*/*v*) in isotonic 100 mM potassium phosphate buffer (pH7.4) containing 1 mM EDTA and 0.1 mM phenylmethylsulfonylfluoride (PMSF, a protease inhibitor). The homogenate was centrifuged at 1000*g* for 10 min and supernatant was used for further analysis. Protein concentration was measured using BioRad reagent as described before [15–18].

### 3.3. Measurement of ROS

Production of ROS in ZDF and ZL rat heart cellular fractions was measured using the DCFDA fluorescence method as described before [18].

### 3.4. Protein and Lipid Peroxidation (LPO) and Catalase Activities

Protein peroxidation as a marker of increased oxidative stress was measured in ZDF and ZL rat hearts by DNPH conjugation method as described before [18]. NADPH-dependent-membrane lipid peroxidation was measured as malonedialdehyde formed using the standard thiobarbituric acid method as described before [15]. Catalase activity was performed by the method of Beers and Sizer (1952) in which the disappearance of peroxide was followed spectrophotometrically at 240 nm. One Unit decomposes one micromole of hydrogen peroxide per minute at 25 °C and pH 7.0 under the specified conditions [19].

### 3.5. Measurement of GSH-Redox Metabolism

GSH is the most important cellular antioxidant protecting tissues from oxidative insults. Alterations in GSH-redox metabolism by GSH-peroxidase/reductase and transferases are the key indicators of perturbed antioxidant metabolism. GSH concentration in the tissue homogenate, prepared as described above, was measured by NADPH-dependent GSSG-reductase catalyzed conversion of oxidized GSSG to GSH. Glutathione *S*-transferase (GST) activity using CDNB, glutathione peroxidase (GSH-Px) activity using cumene hydroperoxide and glutathione-reductase activity using GSSG/NADPH as the respective substrates were measured by standard protocols as described before [15,16].

### 3.6. Measurement of CYP 2E1 Activity

CYP 2E1 enzyme activity was measured in left ventricle homogenate from the ZDF and ZL rat heart using dimethylnitrosamine (DMNA) substrate in the presence of NADPH in the appropriate buffer (pH 7.4) as described before [16].

### 3.7. Measurement of Mitochondrial Respiratory Enzyme Complexes

The freshly isolated heart muscle homogenate (5 μg protein) was suspended in 1.0 mL of 20 mM KPi buffer, pH 7.4, in the presence of the detergent, lauryl maltoside (0.2%). NADH-ubiquinone oxidoreductase (Complex I), and cytochrome c oxidase (Complex IV) activities were measured using the substrates coenzyme Q2 and reduced cytochrome c, respectively, according to previously described methods [18].

### 3.8. SDS-PAGE and Western Blot Analysis

Homogenates (50 μg protein) from ZDF and ZL rat hearts were electrophoretically separated by 12% SDS-PAGE [22] and transferred on to nitrocellulose paper [23]. The expression of specific oxidative stress marker proteins (HO-1, iNOS, CYP2E1, Cyt c Ox) and cell signaling transcription regulatory proteins (JNK, p-JNK and IκB-α) was checked by immunoreactions with their specific antibodies by Western blot analysis as described before [15–18]. β-actin was used a loading control. Densitometric analysis of the protein bands was performed using a gel documentation system (Vilber Lourmat, France) and expressed as relative intensity (R.I) compared to the protein expression of ZL which was arbitrarily taken as 1.0.

### 3.9. Statistical analysis

Values were calculated as mean ± S.E.M. of at least three determinations. Statistical significance of the data was assessed using Student’s *t*-test and *p*-values ≤ 0.05 were considered significant.

## 4. Conclusions

Our results have demonstrated increased oxidative stress and mitochondrial dysfunctions in elderly Zucker diabetic fatty rat hearts. The study has implications in elucidating the etiology and pathology of cardio vascular complications in diabesity.

## Abbreviations

CYP4502E1: cytochrome P450 2E1
DCFDA: 2′,7′-dichlorofluorescein diacetate
DMNA: dimethylnitrosamine
GSH: glutathione
GST: glutathione *S*-transferase
GSH-Px: glutathione peroxidase
GSSG-reductase: glutathione reductase
Cyt c ox: cytochrome c oxidase
LPO: lipid peroxidation
ROS: reactive oxygen species
SDS-PAGE: sodium dodecylsulphate polyacrylamide gel electrophoresis
ZDF: Zucker diabetic fatty
ZL: Zucker lean
